# Risks of long-term port use in enzyme replacement therapy for lysosomal storage disorders

**DOI:** 10.1016/j.ymgmr.2018.02.007

**Published:** 2018-03-07

**Authors:** Christian J. Hendriksz, Paul Harmatz, Roberto Giugliani, Jane Roberts, G. Suren Arul

**Affiliations:** a Salford Royal Foundation NHS Trust, Manchester, UK; b University of Pretoria, Department of Paediatrics and Child Health, Steve Biko Academic Unit, Pretoria, South Africa; c UCSF Benioff Children's Hospital Oakland, Oakland, CA, USA; d Medical Genetics Service, HCPA, Department of Genetics, UFRGS and INAGEMP, Porto Alegre, Brazil; e St. Mary's Hospital, Manchester, UK; f Birmingham Children's Hospital, Birmingham, UK

**Keywords:** Mucopolysaccharidosis, Enzyme replacement therapy, Totally implantable venous access device, Lysosomal storage disorder

## Abstract

Totally implantable vascular access devices (TIVADs) are commonly used in conjunction with enzyme replacement therapy (ERT) for lysosomal storage disorders (LSDs). This case series describes potential complications associated with long-term TIVAD use, such as compromise of skin integrity, infection, or port failures. Best practices and skilled specialists are essential for minimizing complications from long-term TIVAD use for ERT.

## 1 Introduction

A totally implantable vascular access device (TIVAD) is an implanted reservoir with a self-sealing silicone septum attached to a silicone catheter that is tunneled under the skin with its tip positioned in a large central vein. Local anesthetic creams, such as lidocaine/prilocaine (EMLA®) or tetracaine (Ametop®), are applied to reduce pain and minimize trauma during Huber needle accessing.

TIVADs were designed for temporary use, but currently, these devices have been utilized for enzyme replacement therapy (ERT) in lysosomal storage disorders (LSDs), including mucopolysaccharidosis (MPS) disorders, which are lifelong diseases requiring ongoing treatment. Complications of insertion, such as bleeding or bruising, or line dislodgment, break, or blockage, and complications related to long-term usage, such as infection and venous thrombosis, must be considered [[1], [2], [3], [4]]. Additional serious, rare complications, including pneumothorax, hemothorax, pericardial effusion, or cardiac arrhythmias are also possibilities [3].

The purpose of this case series is to discuss the potential risks when considering long-term port use for ERT administration.

## 2 Materials and methods

Individual cases representative of the spectrum of TIVAD-associated complications in patients with LSDs were selected for presentation, based on an informal discussion with clinicians from multiple centers. A group of 64 pediatric patients from Birmingham Children's hospital with inherited metabolic disorders, including LSDs and general metabolic disorders was included for a general (acute and chronic) assessment of TIVAD complication rate in rare diseases. An additional 33 pediatric patients from Birmingham Children's hospital, exclusively with LSDs were assessed over the period of January 2007 to December 2014 to determine complications arising from chronic TIVAD use.

TIVADs were inserted into the internal jugular or innominate veins via an ultrasound-guided percutaneous approach [5] with the port implanted on the chest wall (e.g., below and lateral to the nipple). The tip of the line would be positioned in the high right atrium or superior vena cava/right atrium junction.

## 3 Results

A summary of 8 selected cases are presented in Table 1.

**Table 1:** Demographics and clinical characteristics of patients with port use complications.

Case	Disorder	Age (years)	Sex (M/F)	Time with TIVAD	Complication	Removal of TIVAD? (Y/N/reinserted)	Resolution
1	Alpha mannosidosis	18	M	3 years	Skin thinning and discoloration; small bruise over TIVAD site	N	Change from lidocaine/prilocaine cream to ethyl chloride spray
2	MPS II	19	M	2 years, 8 months	Skin atrophy; bruise around TIVAD site; small scab over port	Reinserted	Change from lidocaine/prilocaine cream to ethyl chloride spray
3	MPS VI	20	M	8 months	Skin burn from overuse of tetracaine cream	Reinserted	Change from tetracaine to lidocaine/prilocaine cream
4	MPS I	N/A	N/A	2 years	Infective endocarditis	Y	Valve replacement
5	MPS II	28	M	8 years	Thrombus	Y	Warfarin treatment; after port removal, used peripheral infusions
6	MPS II	29	M	3 years	Port failure; subsequent difficulties placing pacemaker	Y	Successful placement of pacemaker after third complex procedure
7	MPS I	26	F	2 years; 6 months; 5 years; ongoing	Multiple port failures (fourth port in use); collateral vessel interference with angiography	N	Continue with fourth TIVAD; however, distorted vasculature noted
8	MPS VI	27 at time of death	F	1 year; 6 years	Fatal, generalized infection; port was considered infection entry route	–	–

MPS, mucopolysaccharidosis; N/A, not available; TIVAD, totally implantable vascular access device.

Patient #1 had a TIVAD inserted in 2011 for weekly hospital-based ERT for alpha mannosidosis. After 3 years of treatment, along with use of lidocaine/prilocaine cream, the skin was thinned and discolored, and a small bruise developed over the TIVAD site. Thereafter, the lidocaine/prilocaine cream was changed to ethyl chloride spray. No further changes were seen in skin integrity. In 2016, the patient was admitted to his local hospital with a line infection and the TIVAD was removed; subsequent cannulation was unsuccessful and treatment was discontinued.

Patient #2 began ERT by cannulation for MPS II in 2004; the TIVAD was inserted in 2009 for home infusions with the use of lidocaine/prilocaine cream. After 2 years and 8 months of treatment, a bruise developed around the TIVAD site, along with signs of skin atrophy and a small scab over the port. After another 6 weeks, the wound presented with yellow exudate and the port septum was visible. A skin culture revealed *Staphylococcus aureus*. Subsequently, the TIVAD was removed and reinserted, and the lidocaine/prilocaine cream was changed to ethyl chloride spray.

Patient #3 began ERT for MPS VI in 2006; the TIVAD was inserted in 2009 for home infusions with the use of tetracaine cream. However, the parent overapplied the cream (2 tubes) weekly. After 8 months, the patient was admitted to the hospital; the TIVAD was sitting on the chest wall with little skin covering the site, likely due to tetracaine burn from overuse. The TIVAD was removed and reinserted 8 months later. The family was re-educated regarding local anesthetic use, and the therapy was changed to lidocaine/prilocaine cream.

Patient #4 developed infective endocarditis after 2 years of TIVAD use for MPS I (attenuated) and required valve replacement. Limited information was available on this patient.

Patient #5 began ERT for MPS II in 2005; the TIVAD was inserted in 2006 and lasted 8 years, but with intermittent problems. In 2014, the port site was painful, and the patient felt unwell. Patient had a thrombus and was treated with warfarin for 3 months. The port was removed in 2015, and the patient is now managed by peripheral infusions.

Patient #6 has MPS II with a history of mitral valve repair. ERT began in 2007; the TIVAD was inserted in 2008. In 2009, the port was painful and difficult to flush, but the linogram presented as normal. In 2011, the port failed and was removed. In 2013, a pacemaker was deemed necessary, after 2 episodes of syncope in the prior 2 years, but 3 attempts at placement failed due to abnormal vessels. On the final attempt, the pacemaker was successfully placed with additional new equipment and a difficult and lengthy procedure.

Patient #7 began ERT with a TIVAD in 2004, for MPS I (Hurler-Scheie). She had experienced 3 port failures and reinsertions. In 2013, she developed severe acute aortic valve incompetence and needed valve replacement. During an angiogram procedure, her vasculature was found to be distorted, with collateral venous vessels impinging on arterial vessels.

Patient #8 had slowly progressing MPS VI; the TIVAD was inserted in 2005 and replaced 1 year later. In 2012, she presented with repeated episodes of fever. The primary care center was unfamiliar with risks of port use and made the diagnosis of pneumonia. The patient developed a fatal, generalized infection.

### 3.1 Birmingham Children's Hospital Cohort

Patients ranged in age from <3 to 22 years of age, and length of time with a TIVAD ranged from <2 to >10 years. Seven of 64 (10.9%) TIVADs used for weekly ERT were affected by skin integrity complications; 5 devices required removal.

Of the additional 33 pediatric patients, the most common complication was difficult port access following weight gain with ERT initiation. Additional complications included 3 port revisions due to line blockage and 1 early (within 7 days of placement) line infection; no hemopneumothorax or cardiac problems occurred.

## 4 Discussion and conclusions

TIVADs are widely used in cancer and cystic fibrosis, and are considered safe and effective [6,7]. The most common TIVAD-related complications in patients with cystic fibrosis include occlusion, infection, leakage, pain or discomfort, venous thrombosis, stenosis, and port extrusion [7,8]. In patients with cancer, common TIVAD complications include infection, sepsis, thrombosis, skin dehiscence, and pain [6,9,10]. While some overlap exists, the complication profile for TIVAD use for LSDs is unique.

Due to the characteristics of proteins infused and the clinical nature of LSDs themselves, patients with LSD might experience a higher rate of infusion-related reactions than patients treated for other conditions [11]. In a recent review of TIVAD use in patients with inherited metabolic disorders, the main complications were infection and allergic reaction [11]. Patients with LSD may have abnormal vasculature as part of the disease; this may contribute to higher complication rates in these patients [[12], [13], [14], [15]]. An issue among LSD patients is skin integrity (thinning skin, dimpling, bruising) over the TIVAD sites. Skin erosion at the implantation site is usually a rare complication of long-term TIVAD use, with an estimated incidence of 1.67% in patients with average catheter duration of 335 days [16]. We observed a higher rate of skin complications (10.9%), which while easier to manage than some complications, is supportive of skin involvement in LSDs [17].

Because ERT is considered a lifelong therapy, we encourage the use of peripheral intravenous access; thus, it is important to develop nursing and physician access skills to further this objective. Newer topical vascular imaging techniques and percutaneous ultrasound vascular access tools may facilitate placement in very difficult patients [18]. In a pediatric cohort, use of the ultrasound-guided percutaneous technique meant that venous occlusion of the great veins (internal jugular, innominate, or superior vena cava) was exceptionally uncommon at <3% [19].Additionally, use of distraction techniques during the procedure can greatly reduce the pain and distress for children and adolescents [20].

To decrease the risk of complications in patients with LSD who are using TIVADs for long-term ERT, we recommend following best practices, such as ultrasound guidance to place central venous catheters [[3], [4], [5],^21^], experienced surgical team and personnel [1,5], hand hygiene and barrier precautions, and regular examination for thrombus via ultrasounds [3,22] and of skin integrity over TIVAD sites. The major long-term risk for patients with a potentially lifelong requirement for venous access is occlusion of the great veins and thus consideration should be made from the start to avoid this occurring by using specialist teams of surgeons with an interest in vascular access [23] and the ultrasound-guided percutaneous approach [19,23]. With these measures and careful placement, monitoring, and confirmation of the need for TIVAD, specialists will be equipped to strive for optimal outcomes with ERT with minimal complications from long-term TIVAD use.
